# Cathepsin K maintains the compartment of bone marrow T lymphocytes in vivo

**DOI:** 10.1002/iid3.412

**Published:** 2021-02-16

**Authors:** Renate Hausinger, Marianne Hackl, Ana Jardon Alvarez, Miriam Kehr, Sandra Romero Marquez, Franziska Hettler, Christian Kehr, Sandra Grziwok, Christina Schreck, Christian Peschel, Rouzanna Istvánffy, Robert A. J. Oostendorp

**Affiliations:** ^1^ Technical University of Munich, School of Medicine, Klinikum rechts der Isar, Department of Internal Medicine III ‐ Hematology and Oncology Laboratory of Stem Cell Physiology Munich Germany; ^2^ German Cancer Consortium (DKTK) Heidelberg Germany; ^3^ Present address: Rouzanna Istvanffy Technical University of Munich, School of Medicine, Klinikum rechts der Isar, Surgery Clinic and Policlinic, Laboratory of Pancreatic Neuropathy and Pain Munich Germany

**Keywords:** cathepsin, cathepsin K, CTSK, hematopoietic stem cells, lymphopoiesis, marrow, microenvironment, niche, stem cells

## Abstract

In this study, we investigated the influence of the loss of cathepsin K (*Ctsk)* gene on the hematopoietic system in vitro and in vivo. We found that cultures with lineage^−^ SCA1^+^ KIT^+^ (LSK) cells on *Ctsk* deficient stromal cells display reduced colony formation and proliferation, with increased differentiation, giving rise to repopulating cells with reduced ability to repopulate the donor LSKs and T cell compartments in the bone marrow (BM). Subsequent in vivo experiments showed impairment of lymphocyte numbers, but, gross effects on early hematopoiesis or myelopoiesis were not found. Most consistently in in vivo experimental settings, we found a significant reduction of (donor) T cell numbers in the BM. Lymphocyte deregulation is also found in transplantation experiments, which revealed that *Ctsk* is required for optimal regeneration of small populations of T cells, particularly in the BM, but also of thymic B cells. Interestingly, cell nonautonomous *Ctsk* regulates both B and T cell numbers, but T cell numbers in the BM require an additional autonomous *Ctsk*‐dependent process. Thus, we show that *Ctsk* is required for the maintenance of hematopoietic stem cells in vitro, but in vivo, *Ctsk* deficiency most strongly affects lymphocyte homeostasis, particularly of T cells in the BM.

## Graphical abstract

This manuscript describes how stromal cathepsin K regulates stem cells in vitro and in vivo. Transplantation experiments show that the main effect of the loss of the cathepsin K gene in vivo is a deregulated lymphocyte homeostasis, particularly the T lymphocyte subsets in the bone marrow compartment, in both autonomous and nonautonomous manners.

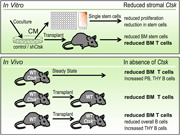

## 1 INTRODUCTION

Secreted proteases regulate hematopoiesis through cleavage of microenvironmental factors such as critical cytokines (KITL/SCF, TGFβ) and chemokines (CXCL12), as well as matrix components and cell surface molecules required for cell–cell interactions.^1^ As such, proteases are instrumental in releasing hematopoietic stem cells (HSCs) from their environment, as in for instance HSC mobilization induced by granulocyte colony‐stimulating factor treatment.^2^ Proteases include cell surface‐bound dipeptidyl peptidases (including CD26 and CD143), metalloproteases (such as MMP9 and ADAMs), and cathepsins.^3^ The lysosomal cysteine protease cathepsin K (gene *Ctsk*, protein: CTSK) is mainly secreted by osteoclasts and cleaves collagens, osteonectin and fibrinogen, thus playing a crucial role in bone resorption.^4^ Deregulation of CTSK may result in osteoporosis by overactivation such as in osteoclast‐mediated bone destruction in bone marrow (BM) tumors as well as in postmenopausal osteoporosis. Inactivation of CTSK by mutations in the *Ctsk* gene are further associated with increased bone density (osteopetrosis) in pycnodysostosis, an inheritable condition marked by skeletal abnormalities.^5^ *Ctsk* is not only expressed by osteoclasts, but it is also found in other cell types such as BM stromal cells.

The role of *Ctsk* expression in BM stromal cells is poorly investigated. We have previously described that *Ctsk* is preferentially expressed by stromal cell lines, which maintain repopulating HSC activity in vitro.^6^ Deletion of other stromal factors expressed by these cell lines, such as *Sfrp1* ^7^ or *Wnt5a*,^8^ results in an increased fraction of HSCs showing cell cycle progression with loss of HSC self‐renewal.^9^ The physiologic role of CTSK in stromal cells of the hematopoietic niche has, so far, not been explored. Considering that *Ctsk* is overrepresented in HSC‐maintaining stromal cell lines, we hypothesized that loss of *Ctsk* may affect HSCs and their repopulating activity in culture. To study this hypothesis, we investigate the effects of knockdown of *Ctsk* in stromal cells in co‐cultures with HSCs. In addition, we explore the physiological role of *Ctsk* in WT and *Ctsk* ^−/−^ mice under steady‐state conditions and in transplantation experiments.

## 2 MATERIALS AND METHODS

### 2.1 Mice

*Ctsk* knockout mice (*Ctsk* ^−/−^) were produced as previously described.^10^ Insertion of a neomycin resistance gene cassette into the *Ctsk* gene introduces a premature translation stop codon into the open reading frame of the *Ctsk* gene.^10^ *Ctsk* ^−/−^ mice were bred for at least six generations on C57BL/6.J (B6) background. Polymerase chain reaction was performed on genomic tail DNA to validate the genotype. Age‐ and sex‐matched B6 (CD45.2) control mice were obtained from Harlan Laboratories. In extrinsic transplantations, C57BL/6.Pep3b.Ptpcr (CD45.1) mice obtained from Taconic served as donors and recipients in intrinsic transplantations. 129S2/SvPasCrl (129; CD45.2) were obtained from Charles River Laboratories. In vitro experiments were conducted with (129xCD45.1) F1 (129xCD45.1) mice.

The animals were kept according to the Federation of Laboratory Animal Science Associations and institutional guidelines. All animal experiments were approved by the Government of Upper Bavaria.

### 2.2 Knockdown of *Ctsk* expression in UG26‐1B6 stromal cells

UG26‐1B6 stromal cells were cultured on 0.1% gelatin coated wells as described.^11^ As previously published,^7^, ^12^ we used established stable *Ctsk* gene knockdowns in stromal cells. In brief, sh*RNA* ^mir^ contructs against *Ctsk*, expressed on the pLKO.1 backbone (Open Biosystems–Thermo Fisher Scientific) were transfected into Phoenix E packaging cell line. Collected virus particles were used for the infection of UG26‐1B6 cells followed by selection with 5 µg/ml puromycin. Two such cell lines are used in this study: *shCtsk*2 and *shCtsk*4. As a control, empty vector backbone pLKO.1 was used.

Successful knockdown of the *Ctsk* gene was validated using Western blot and quantitative PCR. For Western blots, total stromal proteins were separated on SDS‐polyacrylamide gels and transferred to BioTrace polyvinylidene difluoride membranes. After transfer, unspecific antibody binding was blocked, and the membrane was incubated with polyclonal anti‐CTSK or antiactin antibodies (see Table S1). Binding of these two antibodies was visualized using horse radish peroxidase‐conjugated secondary antibody, chemo‐luminescent substrates (SuperSignal West reagents, Thermo Fisher Scientific) and photographic development using X‐ray films.

For quantitative PCR, total stromal RNA was reverse‐transcribed into complementary DNA. Specific *Ctsk* sequences were then amplified using primers described in Table S2. Amplicons were either visualized for genotyping on 1% agarose gels, or by quantifying fluorescent SYBR Green incorporation during amplification in a StepOnePlus Real‐Time PCR system (Thermo Fisher Scientific).

### 2.3 Cocultures of stromal cells with HSCs

For cocultures, UG26‐1B6 derivatives sh*Ctsk2 and ‐4*, and pLKO.1 were grown in six well plates to 90% confluence and irradiated with 30 Gy. 1 × 10^4^ lineage^–^ (Lin^−^) BM cells were plated on stromal cells and cultured in long–term culture medium (M5300; Stem Cell Technologies) supplemented with hydrocortisone in a 1:1000 ratio, penicillin/streptomycin and GlutaMAX (200 mM, GIBCO Thermo Fisher Scientific) in a 1:100 ratio for two to four weeks. Each week, half of the supernatant was replaced with fresh medium. After coculture, cells were either used for colony forming unit assays or for in vivo intravenous transplantation assay.

### 2.4 Hematopoietic colony assays

The colony forming assay was performed with growth factor‐supplemented methylcellulose (MethoCult GF 3434; Stemcell Technologies). A total of 1 × 10^4^ Lin^−^ BM cells were seeded into methylcellulose on two 3.5 cm dishes. After 10 days of culture at 37°C, 5% CO_2_, and more than 95% humidity, the colonies formed were counted under the microscope.

### 2.5 Isolation of BM cells

BM cells were isolated from femora and tibiae of 8‐ to 12‐week‐old mice. The ends of the bones were cut open and the marrow was flushed out and resuspended in HF2+ buffer (Hank's balanced salt solution with 2% FCS and 10 µM HEPES buffer) using a blunt needle, then filtered and centrifuged. To obtain Lin^−^ cells, magnetic selection was performed with a lineage cell depletion kit obtained from Mitenyi Biotec. Further purification was performed via cell sorting (below).

### 2.6 Single cell culture

Serum‐free conditioned medium (CM) was essentially generated as described^8^, ^12^, ^13^ by incubating serum‐free medium (BIT 9500; STEMCELL Technologies) for 3 days on irradiated (30 Gy) and confluent pLKO.1 and sh*Ctsk* stromal cells.

Single cell cultures were performed as previously published.^8^, ^12^, ^13^ Briefly, HSC‐enriched CD34^−^ CD48^−^ CD150^+^ Lin^−^ SCA1^+^ KIT^+^ (CD34^−^ SLAM) cells from the BM were sorted after lineage‐depletion of 8‐ to 12‐week‐old B6 control mice and deposited by FACS sorting as single cells in round‐bottomed 96‐well plates, one cell per well. Each well was filled with 100 µl of 0.22 µm filtered CM, supplemented with 100 ng/ml mSCF and 20 ng/ml interleukin (IL)‐11 (both from R&D Systems). For each condition, one 96‐well plate was used containing 60 replicates per experiment. The outer 36 wells were filled with 200 µl of distilled water to prevent the culture from drying out. The clone size was counted each day under the light microscope.

We previously described that this assay assesses separate control of HSC viability, proliferation, and self‐renewal.^8^, ^12^, ^13^ Cell viability was determined by assessing the number of wells with viable cells, whereas proliferation was determined by comparing the number of cells per well between different days. From these cell numbers, the number of cell divisions was estimated using the Log2 of the number of cells counted. After 5 days, the cells were harvested and analyzed by flow cytometry.

### 2.7 Transplantation assay

Transplantation experiments for repopulating capacity were performed as previously described.^7^, ^8^ All cocultures were set up with Lin^−^ cells from 129xCD45.1 (CD45.1xCD45.2) mice and transplanted into lethally irradiated (9 Gy) 129xB6 (CD45.2) recipients. For transplantation of primary cells, 2.5 × 10^5^ freshly isolated BM donor cells from 8‐ to 12‐week‐old mice were injected into 8‐ to 12‐week‐old, lethally irradiated (8.5 Gy) recipients. In “extrinsic transplantations” (i.e., where *Ctsk* was deleted in recipients), CD45.1 WT donor cells were transplanted into either CD45.2 WT or *Ctsk* ^−/−^ recipients. In “intrinsic transplantations” settings (i.e., where *Ctsk* was deleted in the donor cells), CD45.2 WT or *Ctsk* ^−/−^ BM cells were transplanted into WT CD45.1 recipients.

During the first 5 weeks after transplantation, the recipient mice received antibiotic treatment. Sixteen weeks after transplantation, the mice were sacrificed and their peripheral blood (PB), BM, spleen (SPL), and thymus (THY) were analyzed for engraftment of donor cells using the congenic system (CD45.1/CD45.2). Positive engraftment was defined as more than 1% positive myeloid and more than 1% lymphoid donor engraftment.

### 2.8 Flow cytometry analysis and cell sorting

Staining of cell surface antigens was performed with the antibodies described in Table S3 and S4. The cell suspension was incubated in HF2+ buffer for 15 min on ice in the dark. Gating strategies for analyses of hematopoietic subpopulations were performed as described previously.^7^, ^8^

Total cell numbers were calculated by multiplying the subpopulation fraction with the total cell number (determined by trypan blue staining and counting on a Neubauer cytometer) of the spleen and thymus. The total BM cell number was estimated by assuming that two femora and two tibiae represent 20% of the total BM.^14^

**Figure 1:**
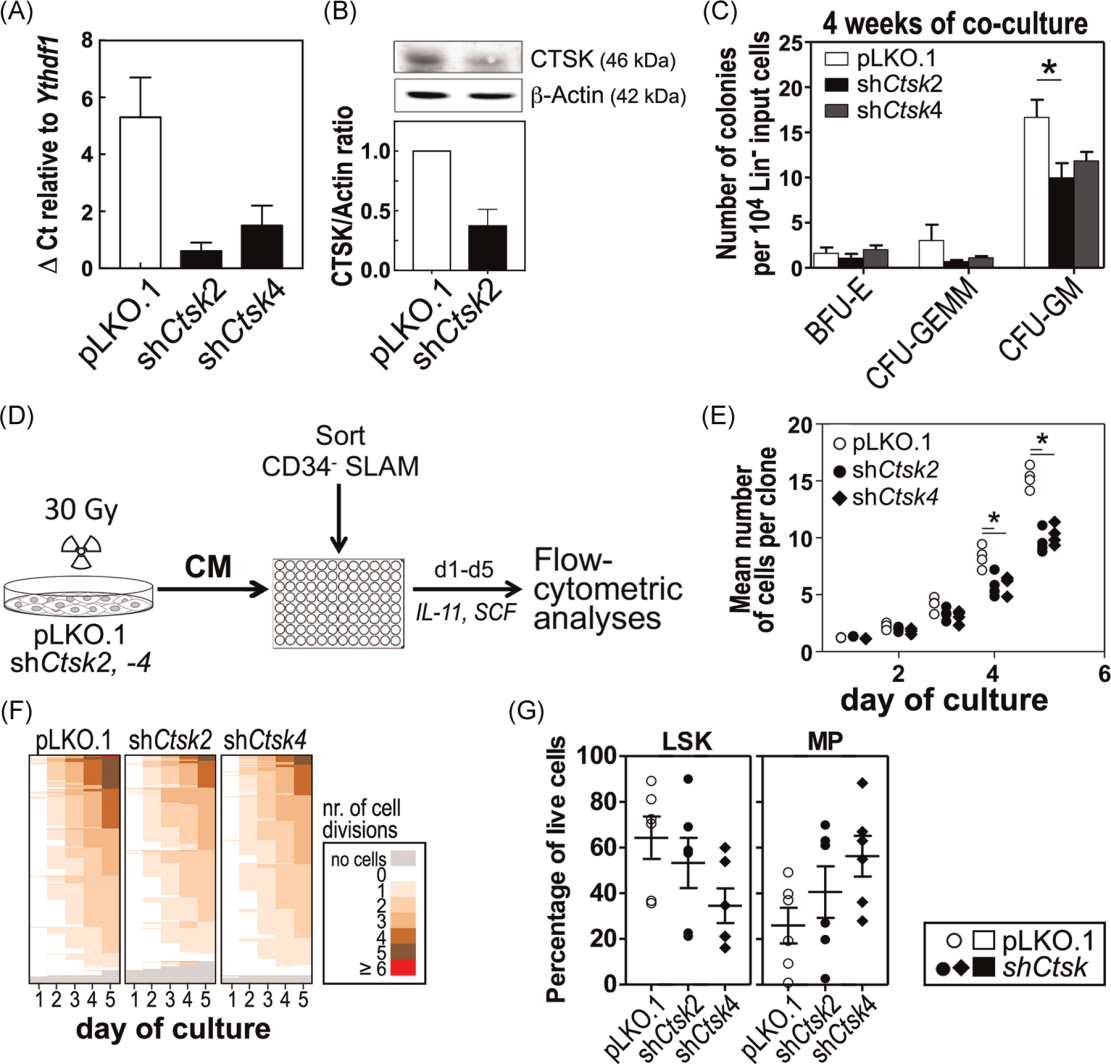
In vitro experiments with UG26‐1B6‐pLKO.1 and ‐sh*Ctsk* knockdown stromal cells. (A) Relative mRNA level, measured by real‐time PCR (*n* = 3) using *Ythdf1* as housekeeping gene, comparing the relative expression of *Ctsk* in the knockdown clone of the UG26‐1B6 (sh*Ctsk2 and shCtsk4*) cells and in the pLKO.1 empty vector control. *ShCtsk2* was used in the follow‐up experiments, unless otherwise indicated. (B) Western Blot showing the relative CTSK protein content to ß‐ actin in UG26‐1B6‐pLKO.1 and ‐sh*Ctsk2* cells (*n* = 3, quantified by ImageJ). (C) Coculture of 10,000 Lin^−^ BM cells on irradiated *shCtsk* and pLKO.1 stromal cells in 10 cm^2^ round dishes for 4 weeks (*n* = 6). After culture, the cells were seeded in methylcellulose and the colonies were counted after 10 days under the microscope. (D) Experimental design of single cell experiments: conditioned medium was generated as described in the Section 2. Single FACS‐sorted CD34^−^ SLAM cells from the BM were sorted in 96‐well plates with pLKO.1 and *shCtsk2* and *shCtsk4* CM supplemented with SCF and IL‐11 and every 24 h microscopically evaluated for cell number. After 5 days (d1–d5), the clones were harvested and analyzed using flow cytometry (*n* = 2). (E) Mean clone size of CD34^−^ SLAM cells cultured with pLKO.1 and *shCtsk* CM. Shown are the results of four independent experiments. (F) Heat map of a representative experiment showing increase in the number of cells of single clones colored by number of divisions. (G) Content of LSK and MP cells per cultured plate. Cells from all wells were pooled and stained for lineage markers (CD45R[B220], CD4, CD8a, CD11b, and Gr1), SCA1 and KIT. Flow cytometry was performed using a CyAn ADP (Beckman Coulter), and the analyses were performed using FlowJo software. Shown are the mean results ± *SEM* of two independent single cell experiments, each with three 96‐well plates (CD34^−^ SLAM cells from separate mice) per condition. *p < .05 using Mann–Whitney *U* test. Open symbols: cultures with control (pLKO) stroma or CM, closed symbols: cultures with sh*Ctsk* stroma or CM. CM, conditioned medium; IL, interleukin; mRNA, messenger RNA

**Figure 2:**
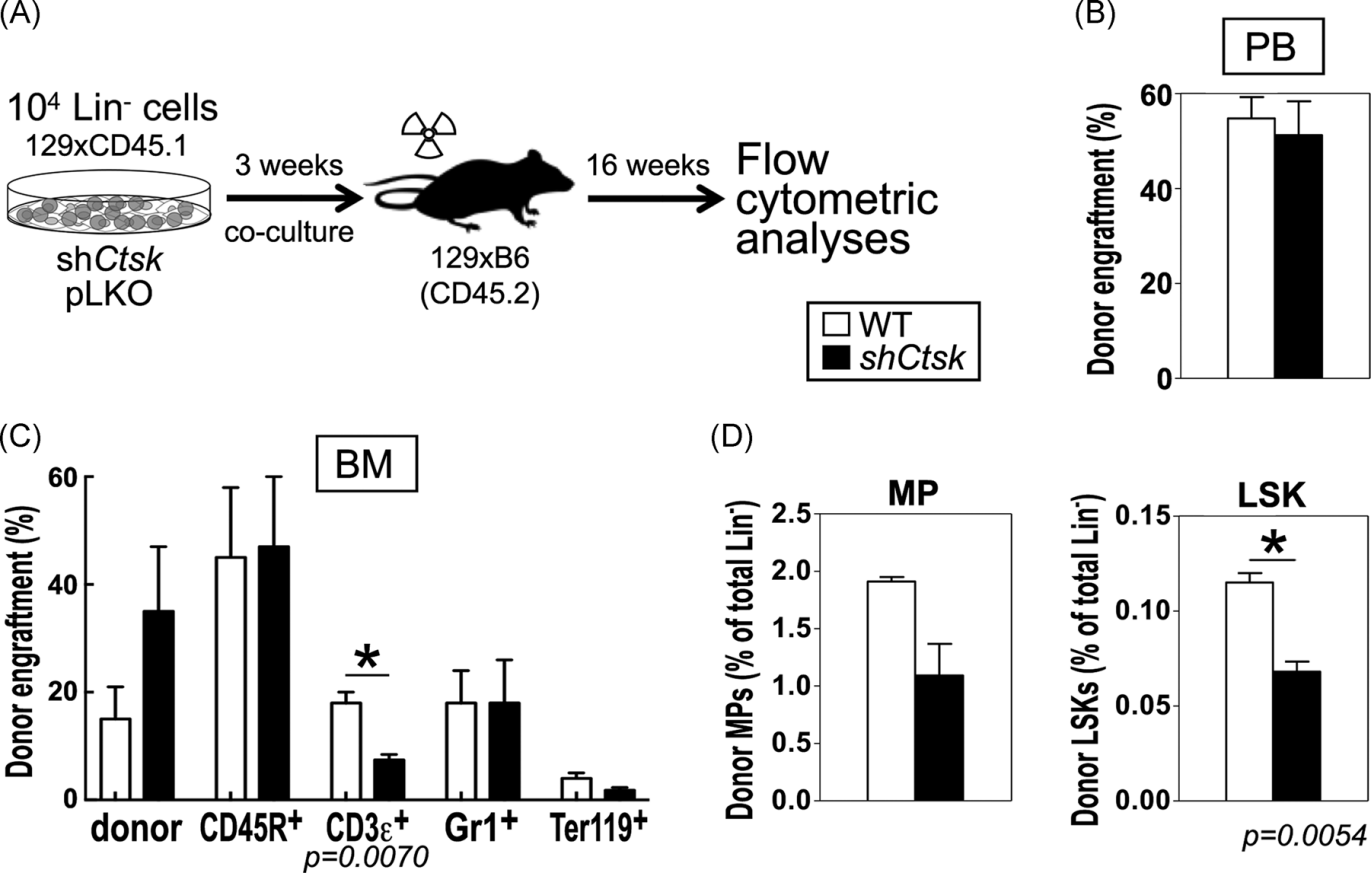
Maintenance of repopulating activity in cocultures of HSCs and UG26‐1B6‐pLKO.1 and ‐sh*Ctsk* knockdown stromal cells. (A) Experimental design of coculture transplant experiments: 10,000 Lin^−^ cells were cocultured on irradiated stromal cells for 3 weeks and then transplanted into lethally irradiated mice (two independent experiments totaling UG26‐1B6‐pLKO: *n* = 7; UG26‐1B6‐sh*Ctsk*: *n* = 6 recipient mice) (B) Flow cytometric analysis of PB, 16 weeks posttransplantation, showing the level of donor engraftment as percentage. (C) Flow cytometric analysis of BM, 16 weeks posttransplantation, showing percentages of donor‐derived mature and (D) early stage hematopoietic cells. Flow cytometry was performed using an EPICS XL (Beckman Coulter), and the analyses were performed using FlowJo software. Shown are the mean results ± *SEM*. *p < .05 using nonparametric Mann–Whitney *U* test. Open bars: cocultures on control stroma; closed bars: cocultures on sh*Ctsk* stroma. BM, bone marrow; PB, peripheral blood

**Figure 3:**
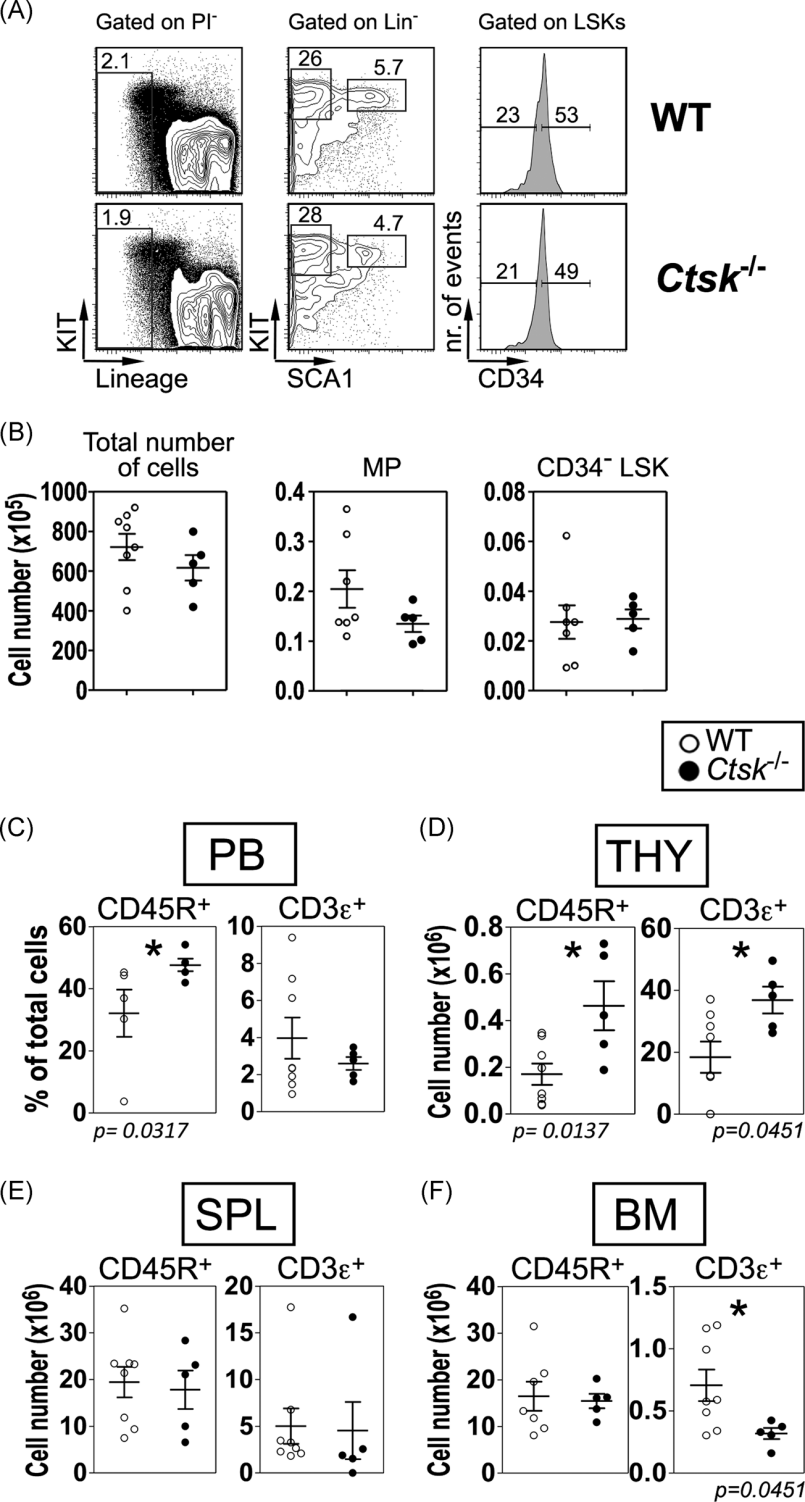
Effects of *Ctsk* loss on steady‐state hematopoiesis. (A) Representative contour plots of the BM stem and progenitor cells from the BM of WT and *Ctsk* ^−/−^ mice. (B) Absolute numbers of cells, myeloid progenitors, and CD34^−^ LSK cells in the BM of WT and *Ctsk* ^−/−^ mice. (C) Percentages of B and T lymphocytes in the PB, and (D) absolute numbers of B and T cells in the THY, (E) SPL, and (F) BM. The data represents results of two to three independent experiments. In (B–E) each dot represents one animal. All flow cytometry was performed using a CyAn ADP (Beckman Coulter), and the analyses were performed using FlowJo software. In each graph, results from individual mice are shown as open (WT control mice) or closed (*Ctsk* ^−/−^ mice) symbols, as well as the mean results ± *SEM*. *p < .05 using the Mann–Whitney *U* test. BM, bone marrow; PB, peripheral blood; SPL, spleen; THY, thymus

**Figure 4:**
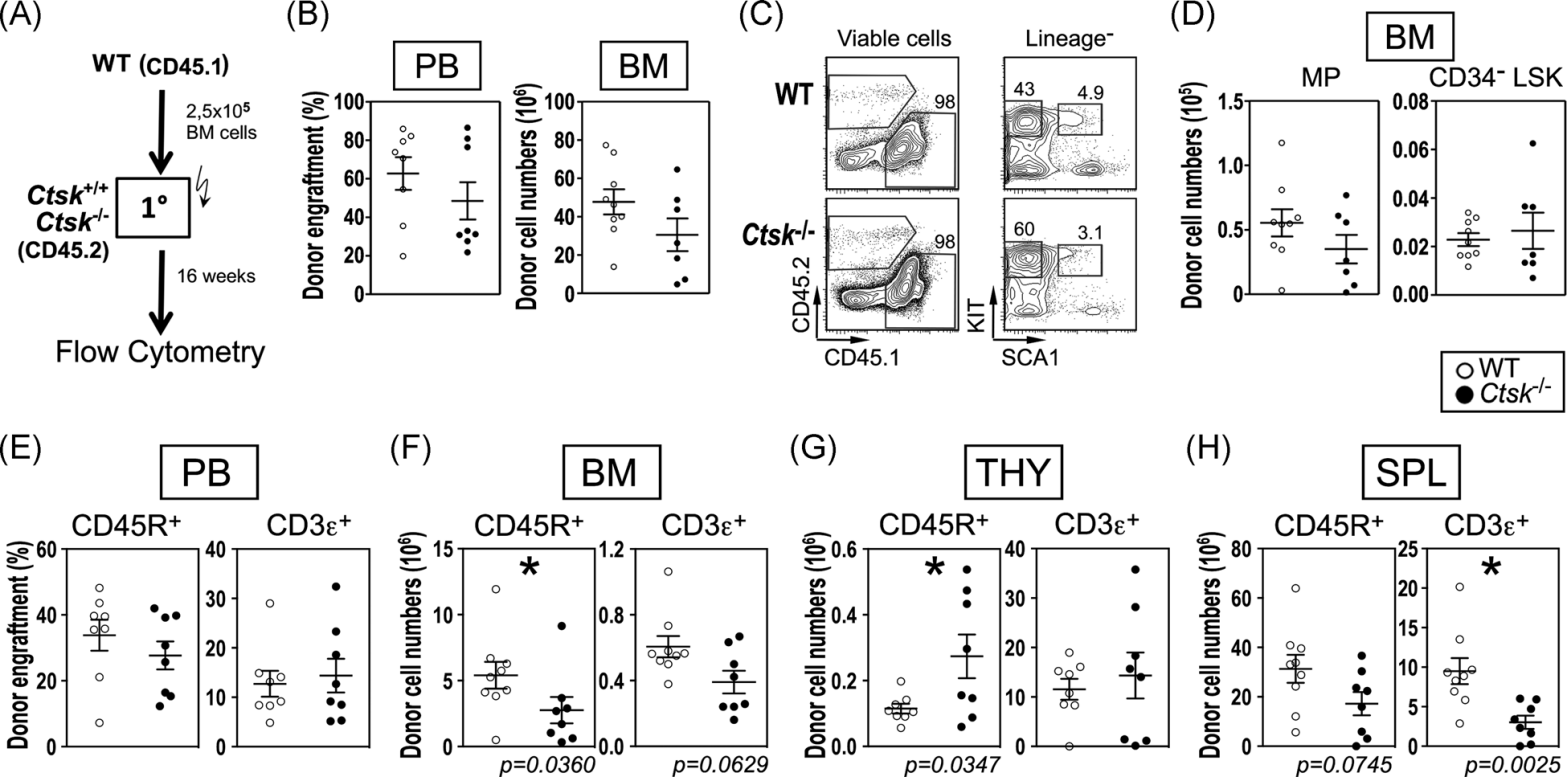
Role of extrinsic *Ctsk* for regeneration of WT HSCs. (A) Experimental design. WT Lin^−^ cells from congenic CD45.1 mice were transplanted into primary (1°) CD45.2 *Ctsk* ^−/−^ mice and their WT littermates. Donor cells were analyzed 16 weeks posttransplantation. (B) Percentage of donor engraftment in the PB. (C) Representative contour plots of the BM stem and progenitor cells. (D) Absolute numbers of MPs and CD34^−^ LSKs in the BM from the extrinsic transplant recipients. (E) Absolute numbers of B and T lymphocytes in the BM, (F) SPL, (G) THY, and (H) SPL. Results of two or three independent experiments are shown. (B and D–G), Each dot plot represents one animal. All flow cytometry was performed using a CyAn ADP (Beckman Coulter), and the analyses were performed using FlowJo software. In each graph, results from individual mice are shown as open (WT control recipients) or closed (*Ctsk* ^−/−^ recipients) symbols, as well as the mean results ± *SEM*. *p < .05 statistically significant using Mann–Whitney *U* test. BM, bone marrow; HSC, hematopoietic stem cell; PB, peripheral blood; SPL, spleen; THY, thymus

**Figure 5:**
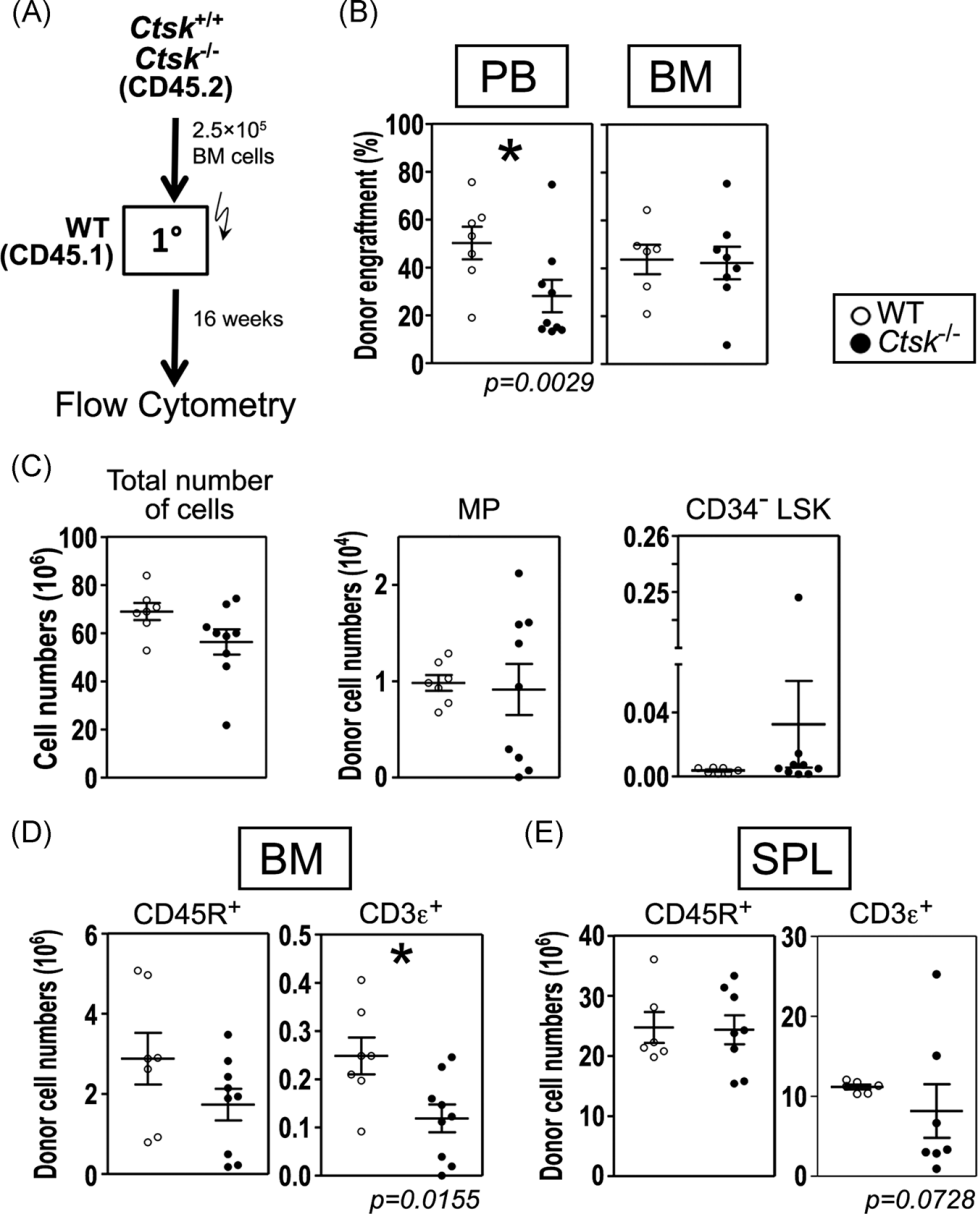
Repopulation capacity of *Ctsk* ^−/−^ HSCs. (A) Experimental design. CD45.2 BM cells from *Ctsk* ^−/−^ and WT control littermates were transplanted into primary (1°) CD45.1 WT recipient mice and analyzed 16 weeks posttransplantation. (B) Percentage of donor engraftment in PB, and BM. (C) Absolute numbers of donor cells, MPs, and CD34^−^ LSKs in the BM of recipient mice. Absolute numbers of donor B and T cells in the BM (D) and SPL (E). The results of two independent experiments are shown. (B–E), Each dot represents one animal. All flow cytometry was performed using a Cyan ADP (Beckman‐Coulter), and the analyses were performed using FlowJo software. In each graph, results from individual mice are shown as open (WT control donors) or closed (*Ctsk* ^−/−^ donors) symbols, as well as the mean results ± *SEM*. *p < .05 statistically significant difference between control and *Ctsk* ^−/−^ donor cells in WT recipients using Mann–Whitney *U* test. BM, bone marrow; HSC, hematopoietic stem cell; PB, peripheral blood; SPL, spleen; THY, thymus

In this study, we used the flow cytometers EPICS XL (Beckman Coulter, Figure 2) and CyAn ADP Lx P8 (Coulter‐Cytomation, Figures 1, 3, 4, and 5) were used for flow cytometric analyses. Data were analyzed using FlowJo software (Tree Star). Results using the two different flow cytometers were not mixed to avoid equipment bias. Cell sorting was performed with a MoFlo High‐Speed cell sorter (Beckman Coulter).

### 2.9 Statistical methods

Normal distribution was examined using the Kolmogorov–Smirnov test, where unclear. Significance levels were determined using the nonparametric Mann–Whitney test. Where more than two groups were compared, we used one‐ way analysis of variance test for analysis of variance with Dunnett's multiple comparison test as post hoc test. All analyses were performed using the software package GraphPad Prism 7 as described in the figure legends. Values with significance level of *p* < .05 are mentioned with a “*” in the figures. Significance levels 0.1 < *p* ≥ .05 are either mentioned as *p* = value in figures, or as *p* < .1 in the text.

## 3 RESULTS

### 3.1 Reduction of stromal cathepsin K impairs proliferation and clonogenic capacity of HSCs in vitro

To study how the absence of stromal *Ctsk* affects hematopoiesis in vitro, we generated *Ctsk*‐knockdowns in the HSC supportive stromal cell line UG26‐1B6 with sh*RNA* ^mir^ (sh*Ctsk2* and sh*Ctsk4*). As control cells, we transduced stromal cells with pLKO.1 empty vector backbone. The *Ctsk* messenger RNA (mRNA) expression was reduced by 70% in sh*Ctsk*4 and down to 90% in sh*Ctsk*2 as compared with pLKO.1 controls (Figure 1A). Also, CTSK protein content was reduced by 63 ± 16% in sh*Ctsk*2 stromal cells (Figure 1B) and by 50% in sh*Ctsk*4 stromal cells (Figure S1A).

To determine whether the reduction of *Ctsk* mRNA affects production of colony‐forming cells, we set up co‐cultures of WT Lin^−^ BM‐cells with pLKO.1 and the *Ctsk‐*deficient stromal cells. These experiments showed that 4 weeks after coculture, the number of granulocytic colony‐forming cells (colony forming unit granulocytes/monocytes: CFU‐GM) was reduced, when stromal *Ctsk* was downregulated (16.7 ± 1.9 for pLKO.1 vs. 9.9 ± 1.6, *p* ≤ .034 for sh*Ctsk2*, and 11.9 ± 1.3, n.s. for sh*Ctsk*4; Figure 1C). To determine whether the decreased production of granulocytic progenitors directly affected HSC‐enriched CD34^−^ SLAM cells, we performed stroma‐free single cell cultures supported by stromal CM from pLKO.1 and sh*Ctsk* stromal cells supplemented with SCF (KIT ligand) and IL‐11 (Figure 1D). In these cultures, cell doubling times and clone sizes of CD34^−^ SLAM cells cultured in either *shCtsk2 or shCtsk4* CM were significantly reduced compared with cells cultured in CM from pLKO.1 control (8.2 ± 0.5 vs. 3.0 ± 0.3; *p* ≤ .001 for sh*Ctsk2* and 5.9 ± 0.4 for sh*Ctsk4*; *p* ≤ .001 on Day 4; Figure 1E,F). Our findings further suggest, that after 5 days of culture, the frequency of the more undifferentiated lineage^−^ SCA1^+^ KIT^+^ (LSK) cells was reduced in cultures with both sh*Ctsk* CMs (34.5 ± 7.6% for sh*Ctsk4*, *p* < .1 and 53.2 ± 10.9% for sh*Ctsk2*, *p* < .1 compared with pLKO.1 CM (64.3 ± 9.3%). In addition, the more differentiated myeloid progenitors (MPs) frequency was increased 1.2‐ to 2.3‐fold after culturing in CM from both sh*Ctsks*; as with the LSKs, these findings did not reach significance (56.2 ± 8.9% for sh*Ctsk*4, *p* < .1 and 40.6 ± 11.3% for sh*Ctsk*2, *p* < .1) compared to pLKO.1 CM (25.9 ± 7.8%; Figure 1G).

To analyze whether the decreased proliferation and the reduction of undifferentiated cells in culture also affected cells with repopulating ability, we transplanted 3‐week cocultures of sh*Ctsk* and pLKO.1 stromal cells and WT Lin^−^ BM cells into WT recipient mice (Figure 2A). Since the sh*Ctsk4* cells were not available for coculture transplantations, we present only data from the sh*Ctsk2* cocultures as sh*Ctsk* cells. Unexpectedly, cells from both types of culture showed similar total engraftment in the PB (Figure 2B). In addition, overall donor myeloid and lymphoid engraftment in the PB were also unchanged between transplants of pLKO.1 and sh*Ctsk2* co‐cultures (not shown). Similarly, donor engraftment in the BM of recipient mice was also unchanged. Interestingly, the only mature population affected after transplantation of Lin^−^ cells cultured on sh*Ctsk* stroma were donor T (CD3ε^+^) cells, which were reduced in the small T lymphocytic BM compartment of recipient mice (17.9 ± 1.6% for pLKO.1 compared to 7.5 ± 0.9% for sh*Ctsk*, *p* = .007, Figure 2C). Furthermore, despite lack of alteration in myeloid populations in the PB, regeneration of the donor HSC‐enriched fraction of LSK cells was significantly reduced in the BM (0.12 ± 0.01% for pLKO.1 compared to 0.07 ± 0.01% for sh*Ctsk*, *p* < .006, Figure 2D).

### 3.2 Loss of cathepsin K modulates in vivo lymphocyte homeostasis

Since reduction of stromal *Ctsk* reduces both number and function of early hematopoietic cells in vitro, we wondered whether *Ctsk* deletion similarly affects hematopoiesis in vivo in *Ctsk* knockout (*Ctsk* ^−/−^) mice, which do not express CTSK protein in the BM (Figure S1B). In 8‐ to 10‐week‐old *Ctsk* ^−/−^ mice we found that early hematopoiesis and myelopoiesis were unchanged under steady‐state conditions compared to WT controls (Figure 3A,B). When evaluating lymphocytic cells (Figure S2), we found that the fraction of peripheral CD45R^+^ (B220^+^ B) cells (32.1 ± 7.6% for WT controls compared to 48.3 ± 2.0% for *Ctsk* ^−^^/−^ mice, *p* ≤ .032) and also the number of the small population of thymic B cells (17.1 ± 0.1 × 10^4^ for WT controls compared to 46.4 ± 0.1 × 10^4^ for *Ctsk* ^−/−^ mice, *p* < .014) were both significantly elevated (Figure 3C,D). Also in the THY, the number of CD3ε^+^ thymocytes was increased (18.4 ± 5.1 × 10^6^ for WT controls compared to 36.8 ± 4.3 × 10^6^ for *Ctsk* ^−/−^ mice, *p* = .045, Figure 3D) with a reduction in the double‐negative CD4^−^ CD8a^−^ T cell population (Figure S1). In contrast, in the SPL, the numbers of B and T cells were neither increased nor decreased (Figure 3E). Interestingly, consistent with the results of transplanted cells from sh*Ctsk* cocultures, we found a small but significant reduction in the modest fraction of CD3ε^+^ (T) cells in four long bones (femurs and tibia) of the BM (70.5 ± 12.6 × 10^4^ for WT controls compared to 31.8 ± 4.4 × 10^4^ for *Ctsk* ^−/−^ mice, *p* = .045, Figure 3F). These results suggest a deregulation in distribution of both B and T lymphocytes over different hematopoietic tissues in the absence of *Ctsk*.

### 3.3 *Ctsk* is required for regeneration of donor lymphocyte numbers

In the next series of experiments, we studied whether the knockout of *Ctsk* in the microenvironment would affect the maintenance of WT HSCs in transplantation settings.^7^, ^8^ For this purpose, we transplanted WT (CD45.1) BM cells into lethally irradiated *Ctsk* ^−/−^ (CD45.2) mice or their WT littermates (Figure 4A). These experiments showed that 16 weeks after transplantation, a comparable peripheral engraftment of donor cells was given (Figure 4B). In contrast to the experiments in which cocultures on sh*Ctsk* stromal cells were evaluated (Figure 2), overall regeneration of HSC‐enriched CD34^−^ LSKs and MPs in the BM of WT and *Ctsk* ^−/−^ recipient mice appeared to be unaffected (Figure 4B–D). When evaluating lymphocytic populations (Figure S3), we found that in accord with unchanged peripheral engraftment, B and T cell populations were also unchanged in the periphery (Figure 4E). However, we found deregulation of donor B and T lymphocyte numbers in several hematopoietic tissues of *Ctsk* ^−/−^ recipient mice as compared with WT littermate recipients. In particular, we found a significant reduction of CD45R^+^ B cells in the BM (5.4 ± 1.0 × 10^6^ for WT recipients compared to 2.8 ± 1.0 × 10^6^ for *Ctsk* ^−/−^ recipient mice, *p* = .036, Figure 4F). In the SPL, the number of donor‐derived CD3ε^+^ cell repopulation was strongly decreased in *Ctsk* ^−/−^ recipient mice (9.5 ± 1.7 × 10^6^ for WT recipients compared to 3.0 ± 0.8 × 10^5^ for *Ctsk* ^−/−^ recipient mice, *p* < .003, Figure 4H), suggesting a role for *Ctsk* in regeneration of the BM and SPL microenvironments *in vivo*. Furthermore, despite reductions in BM and SPL, the number of thymic CD45R^+^ B cells was increased in *Ctsk* ^−/−^ recipient mice (11.4 ± 1.5 × 10^6^ for WT recipients compared to 27.3 ± 6.6 × 10^6^ for *Ctsk* ^−^^/−^ recipient mice, *p* < .035, Figure 4G and Figure S2). These experiments show that *Ctsk* deletion affects optimal repopulation of small lymphoid subpopulations.

To determine whether the lymphocyte deregulation was caused by cell‐extrinsic or cell‐intrinsic loss of *Ctsk*, we transplanted HSCs from *Ctsk* ^−/−^ mice into lethally irradiated WT recipient mice (Figure 5A). Interestingly, 16 weeks after transplantation, we found that *Ctsk*‐deficient BM cells poorly repopulated the periphery of recipient mice (50.2 ± 6.8% for WT donors compared to 28.1 ± 6.8% for *Ctsk* ^−/−^ donor mice, *p* < .003, Figure 5B). However, this reduction in donor cells was not due to impaired hematopoiesis, since engraftment of early donor hematopoietic compartment was unaltered in the BM of recipient mice transplanted with *Ctsk* ^−/−^ cells, suggesting that myelopoiesis was unaffected (Figure 5B,C). With regard to lymphocytic compartments (Figure S4), we found, in accord to extrinsic transplantation results of WT HSCs into a *Ctsk* ^−/−^ environment (Figure 4F), a reduction of the small T lymphocyte fraction in the BM (24.9 ± 3.4 × 10^4^ for WT donors compared to 11.9 ± 2.8 × 10^4^ for *Ctsk* ^−/−^ donor mice, *p* < .016, Figure 5D and Figure S3). However, the SPL (Figure 5E) showed no significant changes in the number of donor‐engrafted lymphocytes.

## 4 DISCUSSION

We explored the role of stromal *Ctsk* in the maintenance of HSCs in vitro and its importance for the regulation of steady‐state hematopoiesis and the number of regenerating lymphocytes in transplantation experiments in vivo. Our experiments show that *Ctsk* expression is required for the maintenance and proliferation of HSCs in vitro and was particularly important for the maintenance of cells regenerating the small fraction of BM T cells. Despite these clear indications of the importance of *Ctsk* for maintaining HSCs in culture, we found that *Ctsk* loss in vivo did not significantly affect HSC maintenance and myelopoiesis *in vivo*. Remarkably, our experiments indicate that *Ctsk* regulates the regeneration of B and T cell numbers in multiple organs. Importantly, while B cell repopulation mainly depends on cell‐extrinsic *Ctsk* expression, the CD3ε^+^‐cell compartment in the BM is consistently affected both by cell autonomous and nonautomonous *Ctsk* deficiency.

We found that knockdown of *Ctsk* in stromal cells reduces their ability to maintain the number and function of HSCs in culture, resulting in reduced production of granulocytic progenitors, lower HSC cell doubling times as well as reduced capacity of maintenance of their ability to repopulate MP and LSK compartments. Considering that CTSK is a cysteine protease, this result suggests that degradation of CTSK substrates is required for maintaining HSCs in culture. One CTSK substrate is collagen. We previously found that collagen‐mediated activation is upregulated in HSCs exposed to stromal factors.^13^ More importantly, together with the neural growth factor, collagen could replace stromal factors to ensure optimal survival of HSCs in culture.^13^ However, here we found no effect of *Ctsk* depletion on the survival‐promoting activity of stromal CM in vitro, suggesting that the specific HSC survival promoting collagen is not affected by *Ctsk*, or that collagen may be substituted by other stromal factors to maintain HSCs in vitro. Other *Ctsk* substrates were shown to be SCF, osteopontin, and the chemokine CXCL12,^3^ which all have documented activity on HSCs and which are not only required for HSC maintenance, but are also involved in HSC trafficking. Displacement of HSCs from the BM requires that the HSCs are released from their microenvironment and that they subsequently transmigrate through endothelial cell layers to gain access to the circulation. There is evidence that *Ctsk* affects both HSC release by regulating the activity of osteoclasts, as well as endothelial transmigration.^3^ However, in previous studies revealing the transcriptome of the HSC‐supportive UG26‐1B6 cells, we did not detect expression of these three factors,^12^, ^13^ suggesting that in cocultures with UG26‐1B6, these three factors are either not expressed at all or at very low levels. Thus, it will now be interesting to determine the relevant *Ctsk* substrates for HSC maintenance on stromal cells in vitro by secretome analysis, and how these are involved in HSC BM and in HSC trafficking.

In contrast to the clear effects of *Ctsk* deletion on HSC proliferation and recovery of immature cells in vitro, the deletion of *Ctsk* only mildly affected early hematopoietic and more mature myeloid cells in the in vivo experimental systems. We originally identified *Ctsk* as being overexpressed in stromal cells supporting HSCs in vitro.^6^, ^15^, ^16^ The over‐represented expression of *Ctsk* was unique amongst the cathepsins, other cathepsins, such as *Ctsa*, *Ctsb*, and *Ctsz* were also expressed but did not distinguish HSC‐supportive and ‐non‐supportive stromal cells.^15^, ^16^ The unique overrepresentation of *Ctsk* in these stromal cells may explain the effects off *shCtsk* knockdown in these cells in vitro. In vivo, *Ctsk* is most highly expressed by mesenchymal sub‐fractions of the hematopoietic niche in the BM.^17^ Although these same cells also express cathepsin B, D, and Z genes, the effects of *Ctsk* deletion may be more difficult to assess, as other cells of the niche, such as osteoclasts and endothelial cells also express *Ctsk*, as well as cathepsins E and S. Considering that osteoclasts^3^ and endothelial cells^18^ are also important in HSC regulation, there may not only be possible redundancy in HSC regulation from cathepsins in the same mesenchymal stromal cells, but also redundancy with cathepsins from nonmesenchymal cells, an aspect we do not model in our coculture system.

In our in vivo experiments, we found a more consistent imbalance in the numbers and distribution of T cells in four different experimental settings in different hematopoietic tissues, particularly the BM. Importantly, we describe deregulation under both steady‐state and transplantation conditions. We found both up‐ and downregulation of T cell numbers under different conditions. Most consistently, we observed a reduction of the number of T cells in the BM in all experimental models explored. The picture is not so clear in other tissues. For instance, under steady‐state conditions, CD3ε^+^ cell numbers in the THY increase significantly with a particular increase in CD4^+^ cells. The mechanism here is unclear but could be caused by decreased T cell selection or reduced cell death. In contrast, under stress conditions, such as regeneration of donor T cell numbers in *Ctsk* ^−/−^ mice transplanted with HSCs, the number of T cells is reduced in the THY, suggesting a differential requirement for *Ctsk* expression under steady‐state conditions and situations of regenerative stress. In this respect, it is interesting to note, that the specific inhibition of cathepsin S, which is coded adjacent to the *Ctsk* gene on mouse Chromosome 3, prevents invariant chain processing and peptide loading required for antigen presentation.^19^ Reduced *Ctsk* activity could, in analogy, lead to dysfunctional presentation of antigens in the context of MHC Class II or CD1d molecules and defective Th2 lymphocyte responses^20^ under stress conditions. In this context, the report that FoxP3^+^ regulatory T cells from *Ctsk* ^−/−^ mice were much more potent in suppressing T effector cells^21^ could also be considered.

The most consistent finding of our study is the reduction of CD3ε^+^ cells in the BM, which is found under steady‐state conditions as well as in transplantation of cultured cells and in both transplantation of WT into *Ctsk* ^−/−^ recipients and vice versa. The regulatory role of the small marrow population of *Ctsk*‐sensitive CD3ε^+^ cells is unclear at the moment. This small cell population in the BM might play an important role in the regulation of hematopoiesis. Indeed, it has been reported that the population of FoxP3^+^ Tregs accounting for 0.15% of total BM, which is slightly less than the number of HSCs,^22^ preserve HSC quiescence and stimulate engraftment.^22^ Together with the above‐mentioned increased potency of Tregs from *Ctsk* ^−/^^−^ mice,^21^ it would be of interest to establish what mechanisms cause the reduction in BM T cells, and particularly FoxP3^+^ Tregs, and how these cells localize and regulate myelo‐ and lymphopoiesis in *Ctsk*‐deficiency.

In addition to imbalances in T cells, we also find in some settings an imbalance in the number of CD45R^+^ B cells in some settings. Under steady‐state conditions, these cells increase in the periphery, and they also increase of 2.7‐fold in the THY. As is the case of the T cell compartment in the BM, B cells represent only a minor population in the THY. Like T cells in the THY, thymic B cells are increased in steady‐state, but they show decreased repopulation in the THY after transplantation. Whereas the reduction of thymic B cells could be caused by impaired regeneration of the thymic microenvironment in the absence of *Ctsk*, the reason for the increase of the thymic B cells under steady‐state conditions remains to be established. This small thymic B cell population has been shown to play a pivotal role in the negative selection of T cells^23^ and it promotes development of regulatory T cells in the THY.^24^ Their development depends on both hematopoietic‐intrinsic and thymic microenvironment‐intrinsic regulatory mechanisms.^25^ Our results suggest that either their development or the homing of B cell precursors into the THY from the periphery is influenced by *Ctsk*, since the total number of B cells combined from the numbers of BM, SPL, and THY B cells is also decreased (Figure S5). It is therefore possible, that in the experiments where we transplanted WT HSCs into *Ctsk* ^−^^/−^ mice, the decline in CD45R^+^ cells in the BM and THY was the result of decreased differentiation into B cells.

In conclusion, *Ctsk* is expressed by stromal cells of the hematopoietic niche. Here we describe that *Ctsk* plays an important role in the maintenance of CD34^−^ SLAM cells and their myeloid differentiation in vitro. In addition, stromal *Ctsk* deficiency decreases the number and function of cells able to regenerate the LSK compartment after transplantation in vivo. Despite these effects of *Ctsk* deficiency, in vitro defects in early hematopoiesis and myelopoiesis are only mild in *Ctsk* ^−/−^ mice, suggesting a redundancy for the requirement of *Ctsk* in vivo which is not observed in vitro. The main effects of *Ctsk* deletion in vivo reveals a recurrent reduction in T lymphocytes in the BM as well as fluctuations of both B and T cell numbers compared to WT animals or recipients in different organs at steady‐state and in transplantation experiments. Importantly, in transplantation experiments, both autonomous and cell nonautonomous regulation by *Ctsk* deficiency can be observed. Thus, our study shows that intact activity of *Ctsk* is required for in vitro HSC maintenance in stromal co‐cultures. In contrast, in vivo, myelopoiesis is only mildly affected by *Ctsk* deficiency, but *Ctsk* maintains B and T cell numbers in different hematopoietic organs, where increases or decreases in cell numbers most probably depend on the effects of stress on the microenvironment. The most consistently observed role of *Ctsk* is to nonredundantly regulate T cell numbers in the BM.

## AUTHOR CONTRIBUTIONS

Renate Hausinger, Marianne Hackl, Ana Jardon‐Alvarez, Miriam Kehr, Sandra Romero Marquez, Franziska Hettler, Christian Kehr, Sandra Grziwok, and Rouzanna Istvanffy performed experiments. Rouzanna Istvanffy and Robert A.J. Oostendorp designed experiments. Renate Hausinger, Marianne Hackl, Ana Jardon‐Alvarez, Miriam Kehr, Sandra Romero Marquez, and Franziska Hettler acquired, and analyzed data. Christian Peschel provided critical infrastructure and reagents. Renate Hausinger, Christina Schreck, Rouzanna Istvanffy, and Robert A.J. Oostendorp interpreted data. Renate Hausinger, Christina Schreck, Rouzanna Istvanffy, and Robert A.J. Oostendorp drafted the manuscript.

## Supporting information

Supporting information.

## Data Availability

Data available on request from the authors.
