# Supplementary material for: Cathepsin K maintains the compartment of bone marrow T lymphocytes in vivo
Source: Immun Inflamm Dis. 2021 Feb 16;9(2):521–32. doi: 10.1002/iid3.412 (PMC8127559; doi:10.1002/iid3.412)
Supplement: Supplementary file 1 — Supporting information. [file IID3-9-521-s001.pdf]

Supplementary files to:

Cathepsin K Maintains The Compartment Of Bone Marrow T Lymphocytes In vivo

Renate Hausinger[1], Marianne Hackl[1], Ana Jardon-Alvarez[1], Miriam Kehr[1], Sandra Romero Marquez[1], Franziska Hettler[1], Christian Kehr[1], Sandra Grziwok[1], Christina Schreck[1], Christian Peschel[1,2], Rouzanna Istvanffy[1,3,*], Robert A.J. Oostendorp[1,*]

Summary

Figure S1 (Additional Western blots to Figure 1)

Figure S2 (Flow cytometry contour plots to Figure 3)

Figure S3 (Flow cytometry contour plots to Figure 4)

Figure S4 (Flow cytometry contour plots to Figure 5)

Figure S5 (Total cell counts Figures 3, 4, and 5)

Table S1. Antibodies used for Western blots

Table S2. Primers used for genotyping and qPCR analyses

Table S3. List of antibodies used for BM cell sorting

Table S4. List of antibodies used for flow cytometric analyses of tissues

Description of cell staining and gating strategies (Materials and Methods supplement)

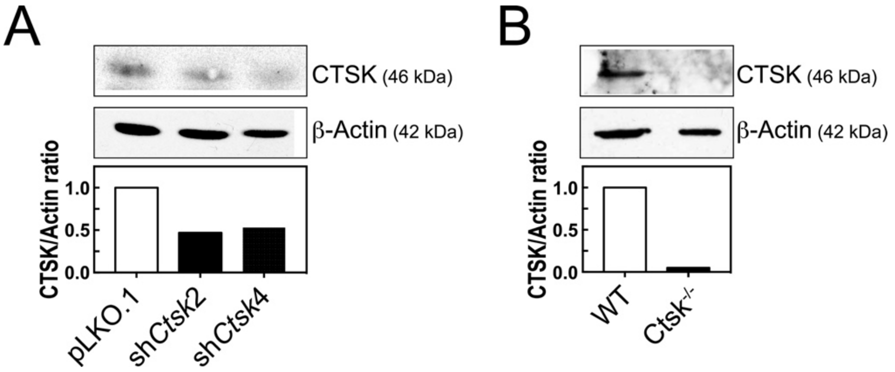

Figure S1 (to Figure 1). Western blot analysis of cell lines and marrow cells used in this study. A. Western blot of the CTSK and β-actin control expression of the UG26-1B6-derived shCtsk lines (sh*Ctsk*2 and -4) and empty vector control (pLKO.1). B. Western blot of the CTSK and β-actin control expression in bone marrow cells from WT and *Ctsk*$^{-/-}$ mice. Western blot was performed as described in the Materials and Methods, Antibodies described in Table S1, total pixels as measure of amount of detected protein measured using ImageJ.

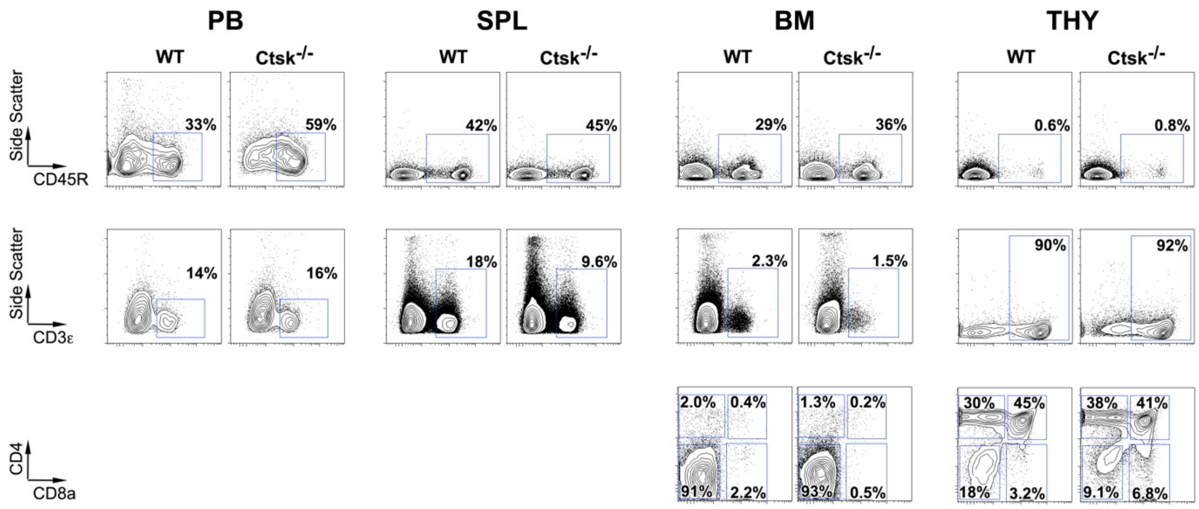

Figure S2 (to Figure 3). Representative flow cytometry contour plots of the PB, SPL, BM, and THY of 8- to 12-week-old WT and *Ctsk*$^{-/-}$ mice under steady-state conditions. Shown is cell staining for CD45R (top panels) and CD3ε (middle panels) for all tissues. For the BM and THY, an additional CD4 and CD8a stain is shown (bottom panels). Wild type samples are shown on the left and *Ctsk*$^{-/-}$ samples on the right hand of each panel. Fluorescence staining was detected using a Beckman Coulter CyAn ADP, and the analyses were performed using FlowJo software.

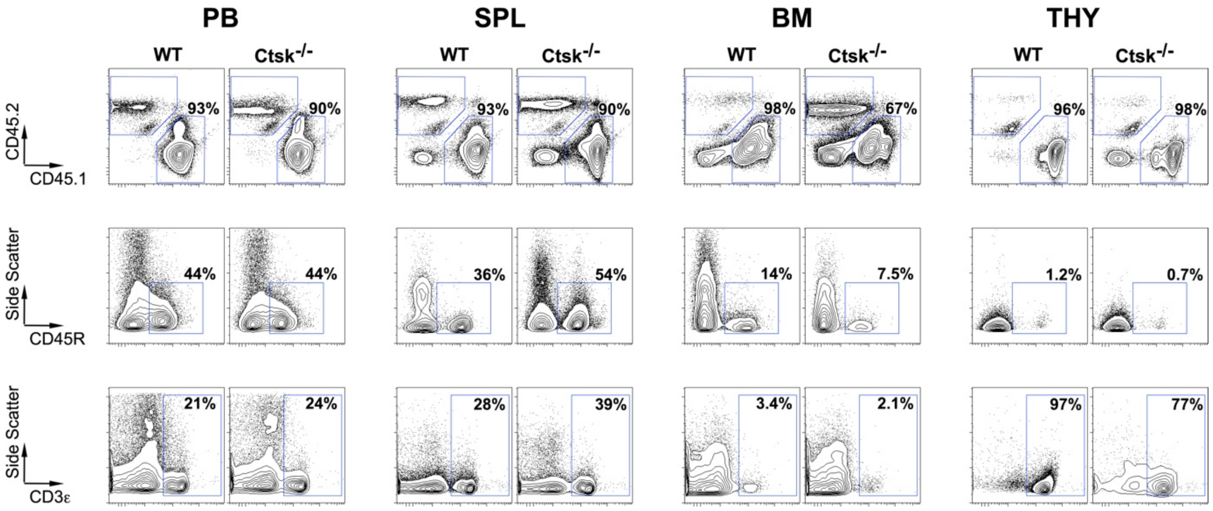

Figure S3 (to Figure 4). Representative flow cytometry contour plots of the bone marrow 16 weeks after transplantation of wild-type CD45.1 donor cells into CD45.2 WT or *Ctsk*$^{-/-}$ recipients. Shown is donor cell staining (CD45.1^{+} CD45.2^{-}, top panels), B cell staining (CD45R/B220), middle panels) and T cell staining (CD3ε, bottom panels) for PB (left panels), SPL (left from middle), BM (right from middle panels, and THY (right panels). Each column represents the different stains and different tissues from the same representative animal. Fluorescence staining was detected using a Beckman Coulter CyAn ADP, and the analyses were performed using FlowJo software.

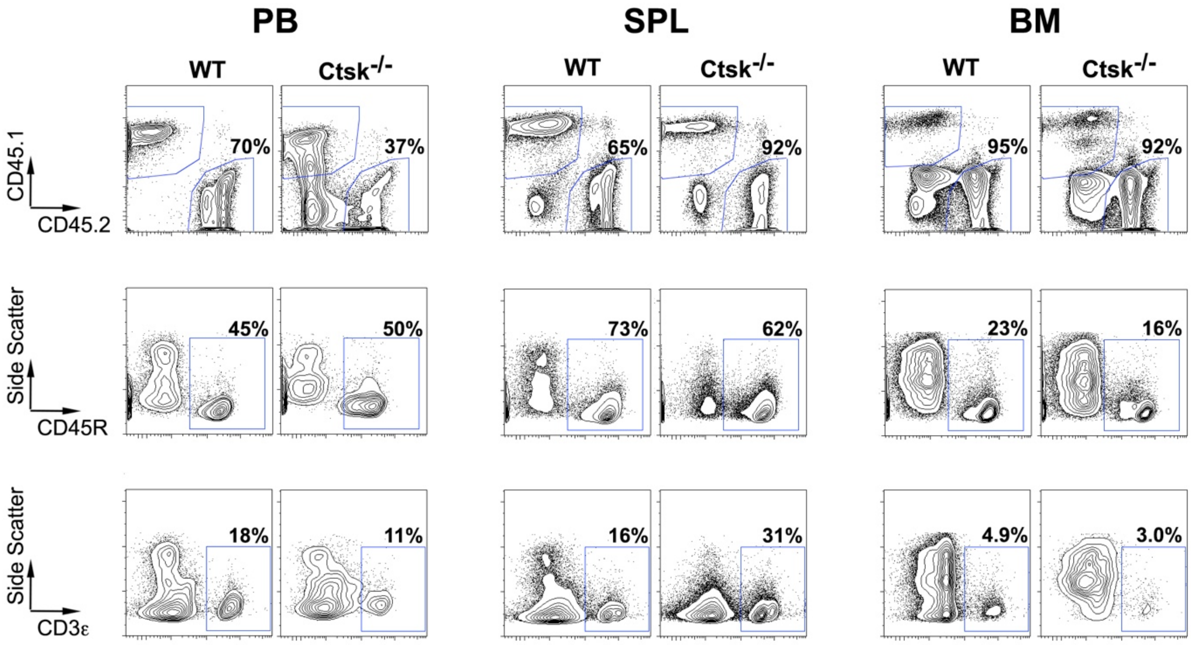

Figure S4 (to Figure 5). Representative flow cytometry contour plots of the PB, SPL, and BM 16 weeks after transplantation of CD45.2 WT or *Ctsk*$^{-/-}$ donor cells into wild-type CD45.1 recipients. Shown is donor cell staining (CD45.1^{-} CD45.2^{+}, top panels), B cells (CD45R^{+}, middle panels), and T cells (CD3ε^{+}, bottom panels) in WT (left columns) or *Ctsk*$^{-/-}$ (right columns) donors. Fluorescence staining was detected using a Beckman Coulter CyAn ADP, and the analyses were performed using FlowJo software.

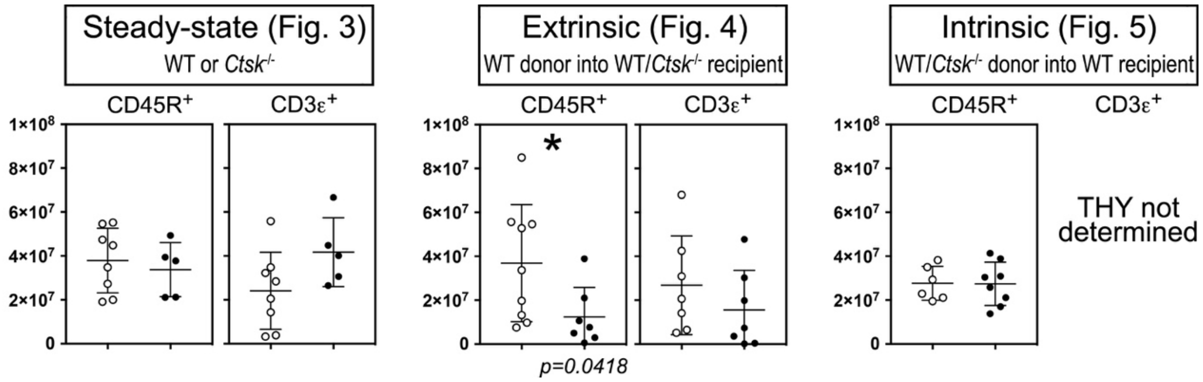

Figure S5 (to Figures 3, 4, and 5). Total cell numbers by combining cell numbers from BM, SPL, and THY, except for the results of the intrinsic transplantations (Figure 5) in which THY cell numbers were not assessed. The cell numbers of PB were not counted, since we could not determine the absolute number of cells circulating in each animal. The left two panels represent the steady-state analyses shown in Figure 3. The middle two panels show the results of the extrinsic transplantation (that is, WT donor cells into either WT or *Ctsk*$^{-/-}$ recipients) from Figure 4. The right panel shows the results of the intrinsic transplantation (that is either WT or *Ctsk*$^{-/-}$ donor cells into WT recipients) from Figure 5. Here, the THY was not analysed in all experiments. The estimates of the total CD45R+ B cells are shown, since the thymic B cell population is negligible compared to the B cell numbers in the BM and SPL. Since the majority of CD3ε+ cells are localized in the thymus, we did not show the total T cell numbers. *: $p<0.05$ statistically significant using Mann-Whitney U-test. Open symbols: WT recipient mice; closed symbols: *Ctsk*$^{-/-}$ recipient mice.

Antigen	Isotype	Clone	Conjugate	Dilution	Manufacturer
Actin	IgG1 (mouse)	#937215		1/10	bio-techne
CTSK (N-20)	IgG (goat)	polyclonal		1/10	santa cruz
Goat IgG	IgG (donkey)	polyclonal	HRP	1/10	bio-techne
Mouse IgG	IgG (sheep)	polyclonal	HRP	1/20	GE Healthcare

Table S1. Antibodies used for Western blot. Conjugate: HRP: horseradish peroxidase.

Target Gene	Exon	Forward	Reverse
CTSK (genomic)	6	GCCACACCCACACCCTAGAAG	ACAAGTGTACATTCCCGTACC
CTSK (total RNA)		ATGTGGGGGCTCAAGGTTCTG	CATATGGGAAAGCATCTTCAGAGTC
ARNT 3 UTR CTSK	1	CTGCTGATGGAAATCTGTTGT	GAGAGAGGGGAAGAGTCGGG
CTSK qPCR	2	CTCCCAGTGGGTGTCCAGTATC	GCTCAAGGTTCTGCTGCTACCT
CTSK qPCR R	3	CCAGTGGGAGCTATGGAAGA	AAGTGGTTCATGGCCAGTTC

Table S2. Primers used for genotyping and qPCR analyses. Genomic and total RNA (for RT-PCR) primers were described by Saftig et al. (Proc Natl Acad Sci USA. 1998; 95: 13453–13458). Primers for qPCR analysis were designed using the Primer3 software and the NCBI nucleotide database.

Antigen	Isotype	Clone	Conjugate	Dilution	Manufacturer
CD3ε	IgG (hamster)	145-2C11	biotin (lineage)	1/10	eBiosciences
CD11b	IgG2b, κ (rat)	M1/70	biotin (lineage)	1/10	eBiosciences
CD34	IgG2a, κ (rat)	RAM34	FITC	1/10	eBiosciences
CD45	IgG2b, κ (rat)	30-F11	FITC, PE, PE-Cy5.5, PE-Cy7, PB, APC, APC-Cy7	1/1	invitrogen
CD48	IgG (hamster)	HM48-1	biotin (lineage)	1/10	invitrogen
CD45R (B220)	IgG2a, κ (rat)	RA3-6B2	biotin (lineage)	1/10	eBiosciences
CD117	IgG2b, κ (rat)	2B8	APC	1/10	invitrogen
CD150	IgG1, κ (mouse)	9D1	PE	1/10	invitrogen
Gr1	IgG2b, κ (rat)	RB6-8C5	biotin (lineage)	1/10	eBiosciences
TER-119	IgG2b, κ (rat)	TER-119	biotin (lineage)	1/10	eBiosciences
SCA1	IgG2a, κ (rat)	D7	PE-Cy7	1/10	invitrogen
Streptavidin			PE-Cy5.5	1/10	Invitrogen

Table S3. Antibodies used for sorting of BM subpopulations. Conjugates: biotin (streptavidin substrate); FITC: fluorescein-isothyocyanate; PE: phycoerythrin; Cy: cyanin; PB: pacific blue; APC: allophycocyanin. For optimal compensation during sorting, fluorescence for

each single dye was set using staining of BM cells with CD45 color controls. Stem cells were sorted for single cell cultures shown in Fig. 1D-G. In this assay, we used the HSC enriched fraction of CD34$^-$ CD48$^-$ CD150$^+$ lineage (CD3ε, CD11b, CD45R, CD48, Ter119)$^-$ SCA1$^+$ KIT$^+$ bone marrow cells.

Antigen	Isotype	Clone	Conjugates	Dilution	Manufacturer
CD3ε	IgG (hamster)	145-[illegible]	PE-Cy5.5, biotin	1/10	invitrogen
CD11b	IgG2b, κ (rat)	M1/70	APC-Cy7, biotin	1/10	invitrogen
CD34	IgG2a, κ (rat)	RAM34	PB	1/10	Invitrogen
CD45	IgG2b, κ (rat)	30-F11	FITC, PE, PE-Cy5.5, PE-Cy7, PB, APC, APC-[illegible]	1/1	invitrogen
CD45.1 (Ly5.1)	IgG2a, κ (mouse)	A20	FITC, PE	1/10	BD Biosciences
CD45.2 (Ly5.2)	IgG2a, κ (mouse)	104	FITC, PE	1/10	invitrogen
CD45R (B220)	IgG2a, κ (rat)	RA3-6B2	PE-Cy7, biotin	1/10	invitrogen
CD117	IgG2b, κ (rat)	2B8	APC-Cy7	1/10	invitrogen
CD150	IgG1, κ (mouse)	9D1	APC	1/10	invitrogen
Gr1	IgG2b, κ (rat)	RB6-8C5	PB, biotin	1/10	invitrogen
TER-119	IgG2b, κ (rat)	TER-119	PE, biotin	1/10	invitrogen
Sca-1	IgG2a, κ (rat)	D7	PE-Cy7	1/10	invitrogen
Streptavidin			PECy5.5	1/10	Invitrogen

Table S4. Antibodies used for flow cytometric analyses of tissues. Conjugates: biotin (streptavidin substrate); FITC: Fluorescein-Isothyocyanate; PE: Phycoerythrin; Cy: Cyanin; PB: Pacific blue; APC: Allophycocyanin. For optimal compensation for analyses, fluorescence for each single dye was set using staining of BM cells with CD45 color controls.

Description of staining and gating strategies *(supplement to the "flow cytometry" section of the Materials and Methods)*

All staining for flow cytometry were performed using single cell suspensions In HF/2+ buffer (see Materials and methods section).

Viable cell staining: In all experiments, viable tissue cells were selected as being negative for the DNA-staining dye propidium iodide (PI$^-$), which, in living cells, does not cross cellular membranes, so that living cells do not stain.

Mature hematopoietic cells were analyzed for positive expression of CD3ε (PE-Cy5.5), CD45R (PE-Cy7), CD11b (APC-Cy7) and Gr1 (PB). The viable cells were then gated for (non-

overlapping) CD3ε^{+} and CD45R^{+} lymphoid populations, and the lymphoid cells were gated for Gr1^{med} CD11b^{+} monocytic and Gr1$^{+/hi}$ CD11b^{+} granulocytic cells.

Immature hematopoietic cells: BM was also stained for immature hematopoietic cells using lineage markers (mix of biotinylated anti-CD3ε, CD11b, CD45R, CD48, and Ter119), CD150 (PE), KIT (APC), SCA1 (PE-Cy7), and CD34 (FITC). The biotinylated mixture of antibodies was visualized using a second staining with streptavidin (PE-Cy5.5).

The viable cells were first selected for Lin- cells (such that in the controls, around 3% of cells did not express any lineage markers). The Lin^{-} cells were then gated for MPs (Kit^{+} Sca1^{-}) and LSKs (Sca1^{+} KIT^{+}). The LSKs were further gated for LT-HSC (CD34^{-} CD150^{+}) and ST-HSCs/MMPs (CD34^{+})

Staining for donor and recipient cells: In transplantation experiments, donor and recipient cells were distinguished using the congenic CD45.1-CD45.2 system using B6/J (CD45.2 WT and *Ctsk*$^{-/-}$ mice) and B6/SJL (CD45.1) mice. For flow cytometric analyses, donor cells were always stained with PE-conjugates and recipients with FITC-conjugates of the antibodies against the two CD45 isoforms. The extra FITC and PE affected the mature cell staining in that the staining for Ter119 was not performed. In staining for immature cells, staining for expression of KIT, CD34, and CD150 was performed using APC-Cy7, PB, and APC conjugates, respectively.
